# Serum immune modulators during the first cycle of anti‐PD‐1 antibody therapy in non‐small cell lung cancer: Perforin as a biomarker

**DOI:** 10.1111/1759-7714.13650

**Published:** 2020-09-11

**Authors:** Takayo Ota, Tomoya Fukui, Yoshiro Nakahara, Takayuki Takeda, Junji Uchino, Takako Mouri, Keita Kudo, Saki Nakajima, Tomohiro Suzumura, Masahiro Fukuoka

**Affiliations:** ^1^ Department of Medical Oncology Izumi City General Hospital Osaka Japan; ^2^ Department of Respiratory Medicine Kitasato University School of Medicine Sagamihara Japan; ^3^ Department of Thoracic Oncology Kanagawa Cancer Center Yokohama Japan; ^4^ Department of Respiratory Medicine Japanese Red Cross Kyoto Daini Hospital Kyoto Japan; ^5^ Department of Respiratory Medicine Uji‐Tokushukai Medical Center Kyoto Japan; ^6^ Department of Pulmonary Medicine Kyoto Prefectural University of Medicine Kyoto Japan; ^7^ Department of Medical Oncology and Respiratory Medicine National Hospital Organization Osaka Minami Medical Center Osaka Japan; ^8^ Department of Clinical Oncology Graduate School of Medicine, Osaka City University Osaka Japan

**Keywords:** Anti‐PD‐1 antibody therapy, non‐small cell lung cancer, perforin, serum biomarkers

## Abstract

**Background:**

Currently used biomarkers for immunotherapy are inadequate because they are only based on tumor properties. In view of microenvironment changes by tumors, host immunity should be considered, which may result in identifying more accurate and easily detectable biomarkers for daily clinical practice. Here, we assessed serum immune‐modulating factor levels for the response to anti‐PD‐1 antibodies during the first cycle in non‐small cell lung cancer (NSCLC) patients.

**Methods:**

Serum was collected from patients with advanced NSCLC treated with nivolumab or pembrolizumab at several time points during the first cycle. We applied the enzyme‐linked immunosorbent assays (ELISAs) and multiplex assays to measure the levels of immune modulators.

**Results:**

A total of 40 patients treated with nivolumab and 26 patients treated with pembrolizumab were studied. By ELISA, serum perforin, but not granzyme B, was measured in all samples. By multiplex assay, 10 immune modulators, including granzyme B, were measured in some, but not all, samples. Serum baseline perforin levels were strongly associated with increased progression‐free survival (PFS) and overall survival (OS) times. Sequential changes in perforin levels during the first cycle were weakly associated with the clinical outcome.

**Conclusions:**

Serum baseline perforin levels may be used to predict the prognosis of NSCLC patients treated with anti‐PD‐1 antibody therapy.

**Key points:**

- To identify a useful predictive marker for anti‐PD‐1 antibody therapy, using blood samples might be helpful.
- Serum baseline perforin levels were closely associated with prognosis with anti‐PD‐1 antibody therapy in non‐small cell lung cancer.

## Graphical abstract

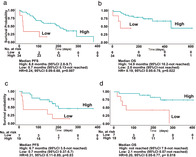

This study demonstrates immune‐modulator changes in serum during the first cycle of anti‐PD‐1 antibody therapy in non‐small cell lung cancer. Serum baseline perforin levels were found to be closely associated with clinical outcome with anti‐PD‐1 antibody therapies.

## Introduction

Cytotoxic lymphocytes (CLs), represented by cytotoxic T cells and natural killer cells, effectively protect against transformed malignant cells or infected cells through the process of cell death, including apoptosis. CLs produce cytotoxic factors to target cells through two direct main mechanisms: granule exocytosis pathways and death ligand‐mediated pathways.^1^, ^2^ Granule exocytosis pathways mediate the exocytosis of secretory granules, such as pore‐forming proteins, perforin, and proapoptotic proteases known as granzymes. Death‐ligand pathways proceed by binding ligands expressed on CLs, which initiate target cell death. Both pathways induce cytotoxicity against cancer cells through apoptosis. Moreover, CL‐mediated cytotoxicity may activate non‐apoptotic pathways to overcome apoptosis resistance.^2^

Programmed cell death 1 (PD‐1) has been previously reported to be isolated from a murine T cell hybridoma and a murine hematopoietic progenitor cell line in which apoptotic cell death is easily induced.^3^ By binding PD‐L1, a ligand of PD‐1, on tumor cells, the PD‐1/PD‐L1 signaling pathway has been shown to inhibit the activation of T lymphocytes, reduce the secretion of antitumor cytokines, and facilitate tumor immune escape.^4^, ^5^, ^6^, ^7^

PD‐1 blockade by anti‐PD‐1 antibodies restores the function of exhausted T cells, reactivates the cytotoxicity of CD8^+^ T cells, and releases perforin, granzyme B, and cytokines, which exhibit cytotoxic activity against tumor cells.^1^, ^5^ We postulated that if patients respond to anti‐PD‐1 antibody therapy, their CD8^+^ T cells respond more actively than those in patients who do not respond. Eventually, the serum concentration of cytotoxic immune modulators may increase more in responders than in nonresponders. Based on this hypothesis, we measured the serum concentrations of perforin, granzyme B, and other immune modulators as biomarkers of the response to PD‐1 blockade in non‐small cell lung cancer (NSCLC) patients. In particular, we focused on the changes during the first cycle of anti‐PD‐1 antibody treatment so that we might be able to identify biomarkers at the earliest time point.

## Methods

### Patients and study design

Patients who were eligible for inclusion in the study had histologically or cytologically confirmed stage IIIB or IV NSCLC. Patients with known epidermal growth factor receptor (EGFR) mutation or anaplastic lymphoma kinase (ALK) translocation were allowed to have received anti‐PD‐1 antibody therapies. Patients had to be 20 years of age or older. Tumor tissue obtained before treatment was used in biomarker analyses. The research protocol was approved by institutional review boards at each participating institution. All participants gave written informed consent before enrollment.

### Study design and treatments

Patients received nivolumab at a dose of 3 mg/kg of bodyweight every two weeks and pembrolizumab at 200 mg per bodyweight every three weeks. Both drugs were administered intravenously. Patients were treated until disease progression or discontinuation of treatment due to unacceptable toxicity, investigator or patient decision to withdraw. Adverse events (AEs) were monitored and graded according to National Cancer Institute Common Terminology Criteria for adverse events, version 4.0. Treatment was interrupted for grade 3 AEs or severe drug‐related AEs until toxicity resolved to less than grade 1.

### Sample collections

Peripheral blood samples were collected into tubes (Terumo Venogect II), mixed well, and then stored for 5–10 minutes at room temperature. Samples were centrifuged at 1000 × *g* for five minutes and stored in 330 μL aliquots at −80°C. Blood samples were collected on days 1, 2, 8, and 15 for nivolumab and on days 1, 2, 8, 15 and 22 for pembrolizumab. For nivolumab, blood samples were collected before nivolumab administration on days 1 and 15. For pembrolizumab, blood samples were collected before pembrolizumab administration on days 1 and 22.

### Assessments

Tumor response was assessed by the Response Evaluation Criteria in Solid Tumors (RECIST), version 1.1. The tumor response was evaluated by a physician every six weeks to three months by using computed tomography (CT). The best overall response was defined as the best tumor response from the start of the treatment to the time of disease progression or death. The best overall response was assessed after three months of follow‐up. The end of the follow‐up period for this study was 31 July 2018.

### PD‐L1 expression analysis

Tumor PD‐L1 protein expression was assessed using immunohistochemistry (IHC) in formalin‐fixed tumor samples at SRL Inc. (Tokyo, Japan). A monoclonal anti‐PD‐L1 antibody 22C3 pharmDx (Agilent Technologies, Tokyo, Japan) was used and stained by an automated stainer (Dako Autostainer Link 48, Agilent Technologies). The stained slides were assessed by pathologists at each institution. The tumor proportion score (TPS) was evaluated on samples with at least 100 viable tumor cells in specimens with membranous PD‐L1 expression. TPS was calculated as the percentage of PD‐L1‐positive tumor cells.

### Enzyme‐linked immunosorbent assay (ELISA)

We analyzed the serum levels of soluble granzyme B, perforin, and soluble CD137 by ELISA according to the manufacturer's protocol. Granzyme B and perforin ELISA kits were purchased from Mabtech, Inc. (Cincinnati, OH, USA). CD137 ELISA kit was purchased from Thermo Scientific Fisher (Raybiotech, Peachtree Corners, GA, USA). All samples were measured in duplicate. For granzyme B and CD137, serum was diluted 1:1. For perforin, serum was diluted 1:4–1:20. The data were calculated using a four‐parameter logistic curve (elisaanalysis.com).

### Multiplex assay

Using a Magnetic Luminex Assay Human Premixed Multi‐Analyte kit (R&D systems, Inc., Minneapolis, MN, USA), the following 10 factors were measured at Filgen (Nagoya, Japan): CD137, PD‐L1, Fas ligand, glucocorticoid‐induced tumor necrosis factor (TNF)‐related protein (GITR), granzyme B, interferon (INF)‐γ, interleukin (IL)‐2, IL‐6, IL‐10, and TNF‐α. A total of 10 serum samples at four‐time points of patients treated with nivolumab and six serum samples at five‐time points of patients treated with pembrolizumab were measured. Serum was diluted 1:1, and all samples were measured in duplicate.

### Statistical analysis

Kaplan‐Meier analysis of PFS and OS was performed based on perforin levels, with differences between each pair of groups being assessed with the log‐rank test. Hazard ratios (HRs) and associated 95% confidence intervals (CIs) were assessed by a Cox proportional‐hazards regression model. All *P*‐values less than 0.05 were considered statistically significant. Statistical analysis was performed using GraphPad Prism, version 8 (GraphPad Software), R (http://www.R-project.org.) or EZR (version 1.42).^8^

## Results

### Patients and treatment with nivolumab

From November 2016 through March 2018, we enrolled 41 patients for nivolumab treatment. One patient was excluded before the treatment started because an *EGFR* mutation was found, and we considered an EGFR tyrosine kinase inhibitor treatment to be preferable. The clinical characteristics of the patients are summarized in Table 1. The median age of the patients was 73 years. Of the 40 patients, 24 (60%) had adenocarcinomas, and 14 (35%) had squamous cell carcinomas. A PD‐L1 tumor proportion score of 1% or higher were reported in 16 (40%) patients. As of 31 July 2018, the median duration of follow‐up was 6.5 months (range 0.1 to 20.3), and 62.5% of the patients were still receiving the treatment. The median number of treatment cycles was 7.5.

**Table 1:** Baseline patient characteristics

	Nivolumab	Pembrolizumab
	*n* = 40	*n* = 26
Median age (years)	73 (39–91)	72 (53–86)
Sex		
Male	30 (75%)	22 (85%)
Female	10 (25%)	4 (15%)
Molecular alterations		
*EGFR* mutation		
Positive	5 (13%)	0 (0%)
Negative	27 (68%)	18 (69%)
Unknown	8 (20%)	8 (31%)
ALK fusion protein		
Positive	1 (3%)	1 (4%)
Negative	29 (73%)	17 (65%)
Unknown	10 (25%)	8 (31%)
Histology		
Squamous	14 (35%)	8 (31%)
Adenocarcinoma	24 (60%)	14 (54%)
Nonsquamous, nonadeno	0 (0%)	3 (12%)
Undifferentiated	2 (5%)	1 (4%)
Previous treatment regimens		
0	0 (0%)	14 (54%)
1	25 (66%)	10 (38%)
2	10 (25%)	2 (8%)
3	4 (10%)	0 (0%)
4	1(3%)	0 (0%)
Smoking status		
Current or former smoker	30 (75%)	23 (88%)
Never smoked	9 (23%)	3 (12%)
Unknown	1 (3%)	0 (0%)
PD‐L1 expression level		
<1%	13 (33%)	
≥1%	16 (40%)	
Unknown	11 (28%)	
≥5%		26 (100%)
<5%		0 (0%)
≥50%		23 (88%)
<50%		3 (12%)

ALK, anaplastic lymphoma kinase; EGFR, epidermal growth factor receptor; PD‐L1, programmed death‐ligand 1.

### Patients and treatment with pembrolizumab

From March 2017 through March 2018, we enrolled 27 patients for pembrolizumab treatment. One patient was excluded from the analysis because of unnatural death. The clinical characteristics of the patients are summarized in Table 1. The median age of the patients was 72 years. Of the 26 patients, 14 (54%) had adenocarcinoma, and eight (31%) had squamous cell carcinoma. A total of 14 (54%) patients were treated with pembrolizumab as first‐line therapy. As of 31 July 2018, the median duration of follow‐up was 6.7 months (range 0.6 to 13.5), and 65.4% of the patients were still receiving the treatment. The median number of treatment cycles was five.

### Measurement of perforin and granzyme B by ELISA

Using ELISA, we assessed serum perforin and granzyme B levels during the first cycle of nivolumab or pembrolizumab in advanced NSCLC. Serum granzyme B was challenging to measure by ELISA. Serum perforin was measured in all samples.

### Multiplex assay

A total of 10 immunomodulators were measured simultaneously using Human Magnetic Luminex Assays. Granzyme B was included in the 10 immunomodulators. Samples that showed different types of efficacy were chosen (Table 2). With nivolumab, 10 cases, and with pembrolizumab, six cases were measured. Out of the 10 markers, only CD137 and Fas ligand were measured in more than 40% of cases for both nivolumab and pembrolizumab at all time points. Due to the small number of cases, the correlation between these two analytes and the response to anti‐PD‐1 antibodies was hard to determine. As there were difficulties in measuring granzyme B by ELISA, granzyme B was also not measured by the Luminex assay.

**Table 2:** Multiplex assay

	Nivolumab (*n* = 10)	PR/SD/PD	Pembrolizumab (*n* = 6)	PR/PD
Immunomodulators ‐no (%)		4/2/4		4/2
CD137	4 (40)	1/1/2	4 (67)	2/2
PD‐L1	1 (10)	1/0/0	2 (33)	2/0
Fas ligand	9 (100)	3/2/4	4 (67)	3/1
GITR	0 (0)	0/0/0	0 (0)	0/0
Granzyme B	1 (10)	1/0/0	0 (0)	0/0
INF‐γ	0 (0)	0/0/0	0 (0)	0/0
IL‐10	0 (0)	0/0/0	0 (0)	0/0
IL‐2	4 (40)	1/1/2	1 (17)	0/1
IL‐6	2 (20)	1/0/1	2 (33)	1/1
TNF‐α	0 (0)	0/0/0	1 (17)	0/1

GITR, glucocorticoid‐induced tumor necrosis factor (TNF)‐related protein.

CD137 levels were measured by ELISA in 20 cases treated with nivolumab and eight cases treated with pembrolizumab. The cases were different from the cases identified by the multiplex assay. Two cases treated with nivolumab and one case treated with pembrolizumab were difficult to measure. Further analysis revealed that CD137 levels were not associated with the efficacy and prognosis (Fig S1).

### Changes in the serum baseline concentration of perforin

**Figure 1:**
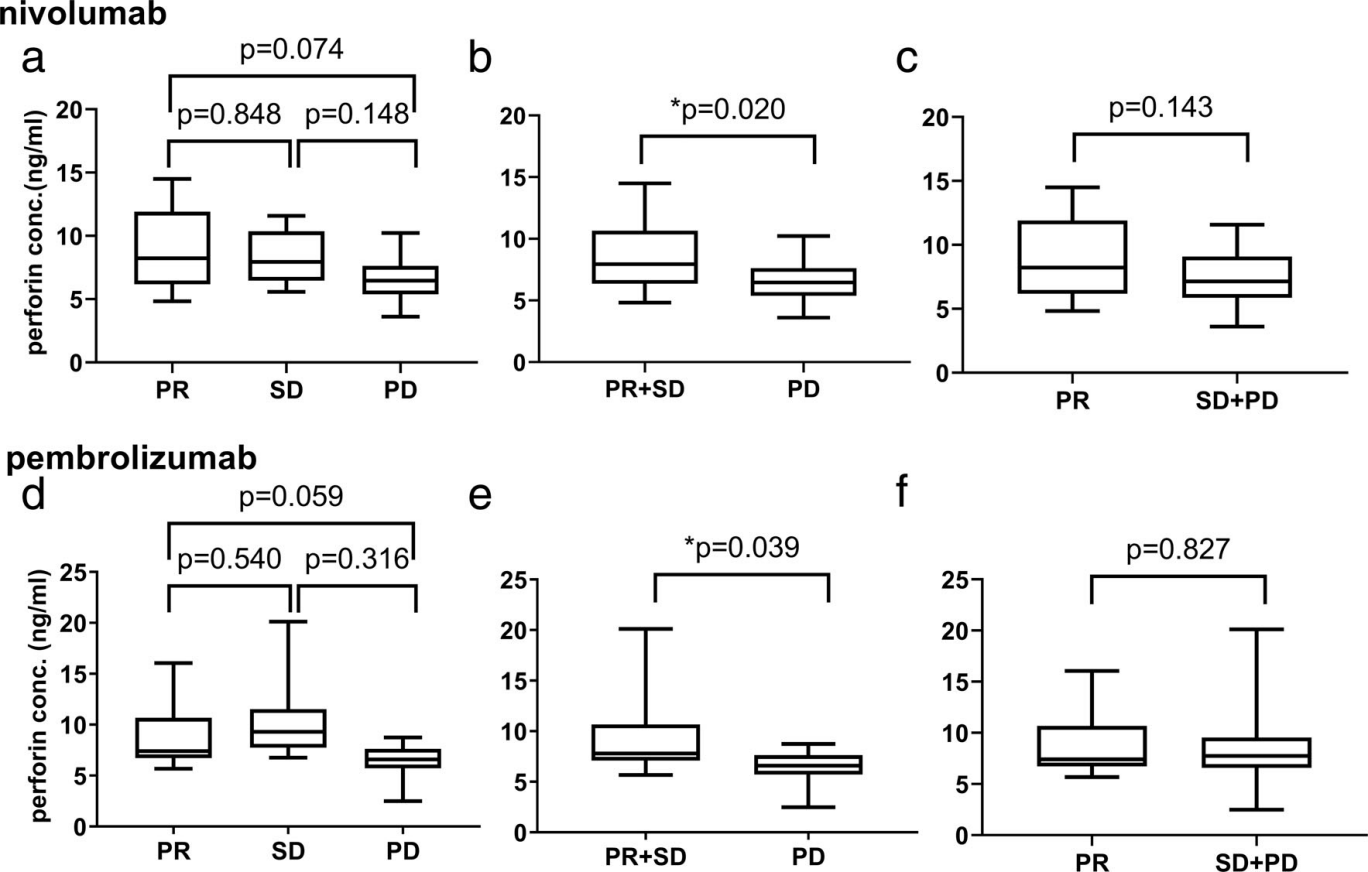
Serum baseline perforin levels and efficacy of nivolumab and pembrolizumab. (**a**, **d**) Baseline levels of serum perforin are plotted, according to RECIST 1.1. (**b**, **e**) PR and SD groups are combined. (**c**, **f**) SD and PD groups are combined. Data were analyzed by analysis of variance (ANOVA) or Student's *t*‐test. The results are the medians, and the whiskers are the minimum to maximum. (**a**–**c**) Nivolumab (PR, *n* = 10; SD, *n* = 15; PD, *n* = 14) and (**d**–**f**) pembrolizumab (PR, *n* = 10; SD, *n* = 8; PD, *n* = 8). * indicates *P* < 0.05.

First, we evaluated the correlation between the baseline concentration and efficacy with perforin levels. In patients treated with nivolumab, we excluded one patient because her target lesion was not assessable due to her death. For partial response (PR), stable disease (SD), and progressive disease (PD), there were no significant differences between groups (Fig 1a). The PR and SD groups were combined, and compared with the PD group, serum perforin levels in the PR and SD groups were significantly higher than those in the PD group (Fig 1b). The SD and PD groups were combined, and compared with the PR group, and there were no significant differences (Fig 1c). For pembrolizumab, we excluded one patient because there were no target lesions to assess. The association between pembrolizumab treatment efficacy and serum perforin levels showed trends similar to those of nivolumab (Fig 1d–f).

Optimal cutoff levels for baseline concentration of perforin were determined by differences in efficacy in PR + SD *vs*. PD. The calculated optimal cutoff levels were 5.45 ng/mL with nivolumab (area under the receiver operating characteristic curve (AUC), 0.703), and 6.63 ng/mL with pembrolizumab (AUC, 0.806) (Fig S2).

**Figure 2:**
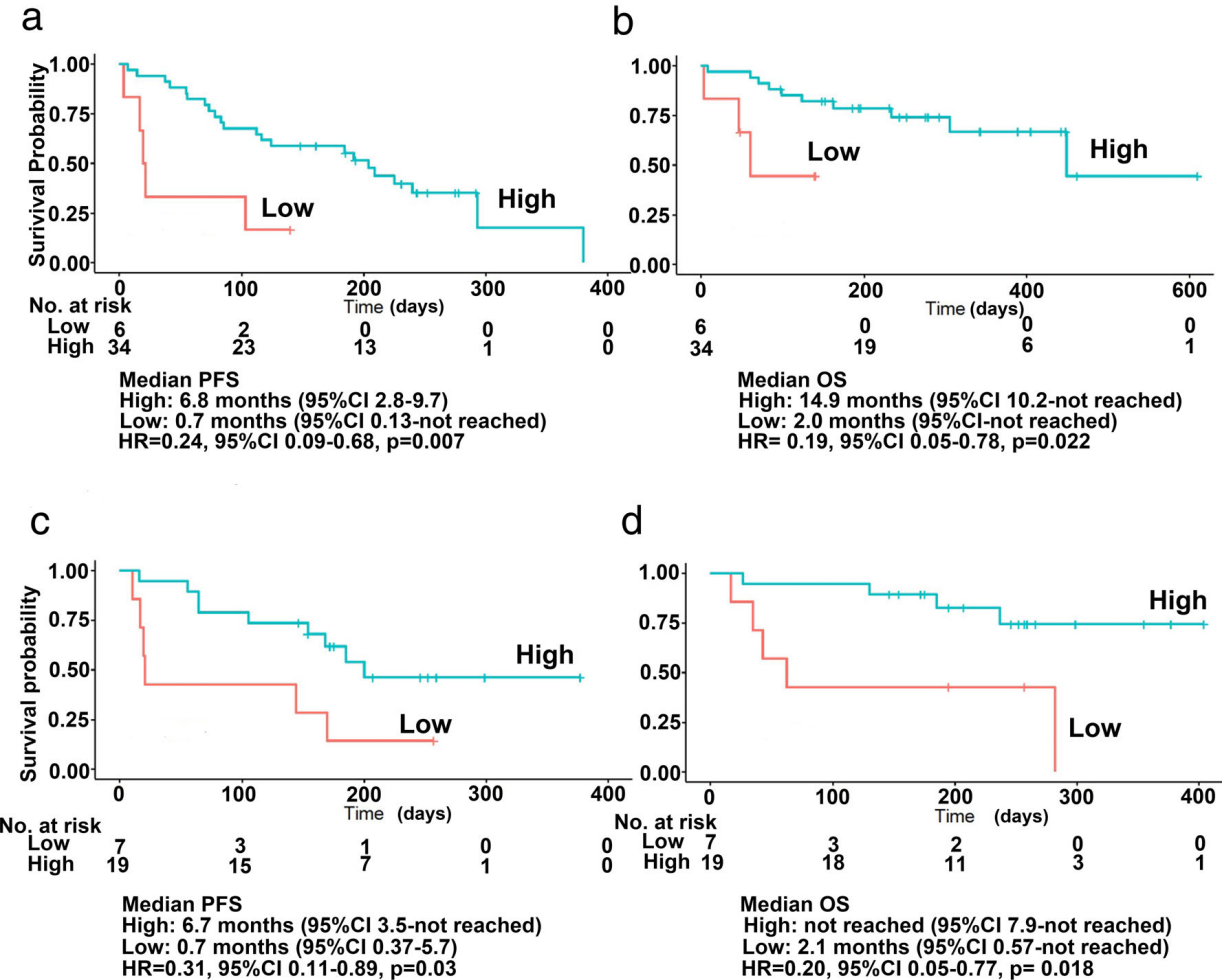
Serum baseline perforin levels and clinical outcomes and the relationship with other markers. (**a**–**d**) Kaplan‐Meier curves for PFS and OS using cutoff levels assessed by PR + SD versus PD. (**a**, **c**) PFS. (**b**, **d**) OS (**a**, **b**) with nivolumab and (**c**, **d**) pembrolizumab.

Using the cutoff levels, cases were separated into high and low concentration groups. The high concentration group was beyond the cutoff value. For patients treated with nivolumab (Fig 2a,b), the median PFS was 6.8 months (95% CI, 2.8 to 9.7) in the high concentration group (85%) versus 0.7 months (95% CI: 0.13 to not reached) in the low concentration group (15%; HR for disease progression or death, 0.24; 95% CI: 0.09 to 0.68, *P* = 0.007). The median OS was 14.9 months (95% CI, 10.2 to not reached) in the high concentration group versus 2.0 months (95% CI: 0.13 to not reached) in the low concentration group (HR for death, 0.19, 95% CI, 0.05 to 0.78, *P* = 0.022).

For patients treated with pembrolizumab (Fig 2c,d), the median PFS was 6.7 months (95% CI: 3.5 to not reached) in the high concentration group (73%) versus 0.7 months (95% CI: 0.37 to 5.7) in the low concentration group (26%; HR for disease progression or death, 0.31; 95% CI: 0.11 to 0.89; *P* = 0.03). Median OS was not reached (95% CI: 7.9 to not reached) in the high concentration group versus 2.1 months (95% CI: 0.57 to not reached) in the low concentration group (HR for death, 0.2; 95% CI: 0.05 to 0.77; *P* = 0.018).

### PD‐L1 expression and perforin levels

**Figure 3:**
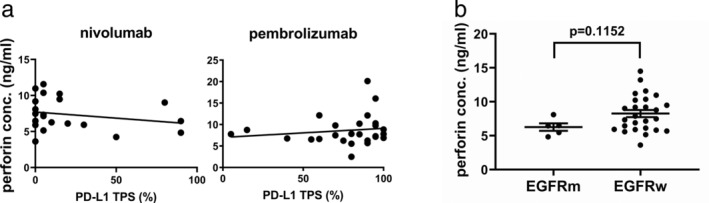
(**a**) Scatter plots of serum baseline perforin levels versus PD‐L1 expression with nivolumab and pembrolizumab. With nivolumab treatment (r squared = 0.022, *P* = 0.4657) and with pembrolizumab treatment (r squared = 0.052, *P* = 0.3052). (**b**) Relationship between serum baseline perforin levels and *EGFR* mutations with nivolumab treatment. Data were analyzed by Student's *t*‐test. The results are mean ± SEM. Perforin conc., perforin concentration; EGFRm, *EGFR* with mutations; EGFRw, EGFR wild‐type.

Because of the reported correlation between PD‐L1 TPS and the response to immune check point inhibitors as anti‐PD‐1 antibody therapy,^9^, ^10^, ^11^ we investigated the PD‐L1 TPS and baseline perforin levels. For both nivolumab and pembrolizumab, there was no correlation between PD‐L1 TPS and serum baseline perforin levels (Fig 3a).

### *EGFR* mutations and perforin levels

In clinical trials, immune checkpoint inhibitors in NSCLC harboring *EGFR* mutation or ALK rearrangements show low efficacy in general.^12^, ^13^, ^14^, ^15^ We evaluated whether *EGFR* mutation or ALK rearrangement is associated with perforin levels. With nivolumab, we observed that perforin levels were lower in *EGFR* mutant cases than in EGFR wild type cases, but the difference was not statistically significant (*P* = 0.1152) (Fig 3b). With pembrolizumab, there were no *EGFR* mutant cases. There were too few patients with ALK rearrangements to analyze the data.

### Sequential changes in perforin levels in advanced NSCLC patients

When exhausted, T cells are activated by anti‐PD‐1 antibody therapy, perforin, granzymes, and other cytokines are released.^1^, ^5^ We hypothesized that immunomodulators are released more and increased the concentrations in the serum with better efficacy or prognosis. The ratios of sequential perforin levels relative to the baseline levels were analyzed, and the optimal cutoff levels determined by efficacy.

**Figure 4:**
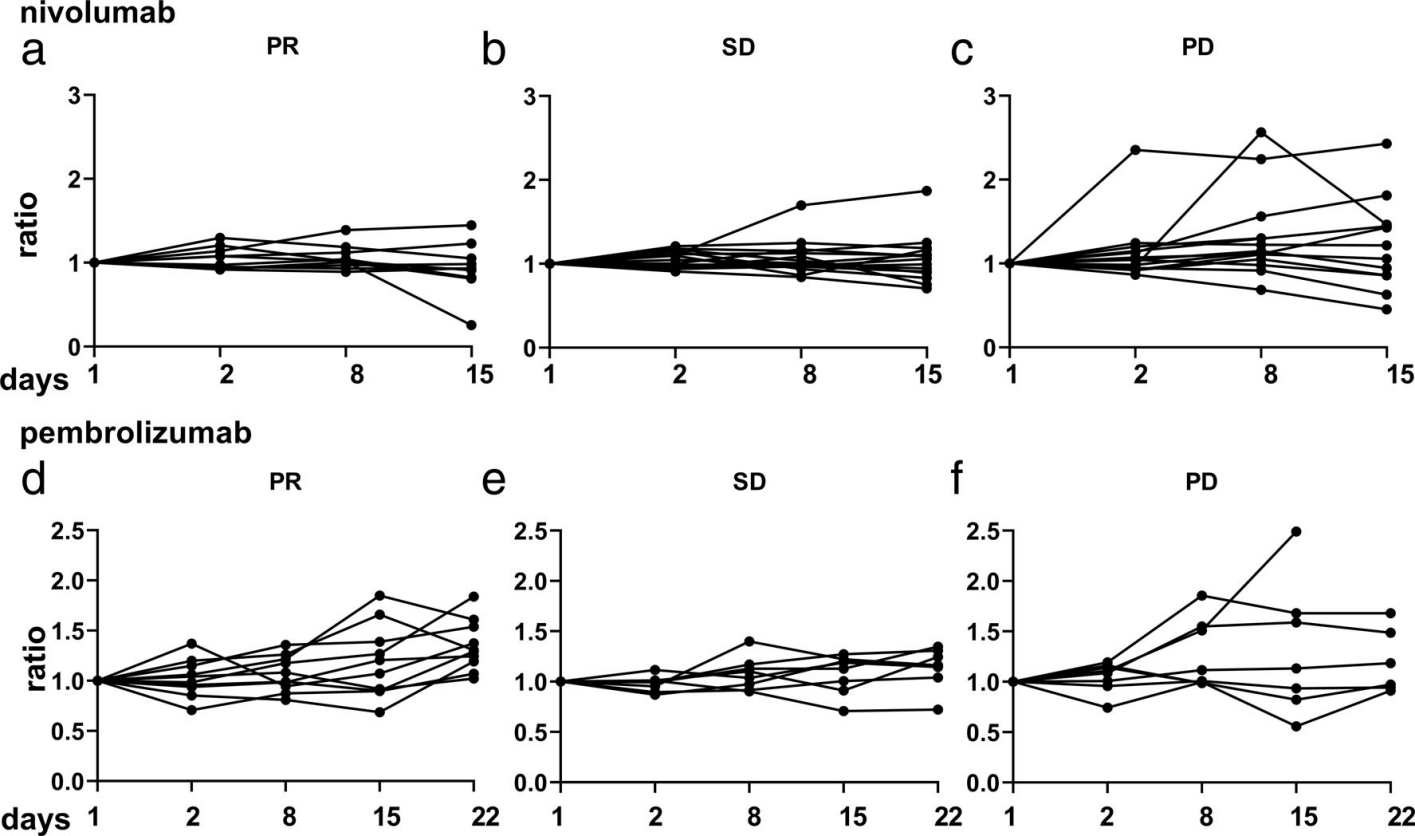
Sequential changes in perforin levels with nivolumab or pembrolizumab treatment. The Y‐axis shows the ratio of the serum perforin concentration at each time point divided by the baseline serum perforin concentration. (**a**–**c**) Nivolumab and (**d**–**f**) pembrolizumab. (**a**, **d**) PR, (**b**, **e**) SD, (**c**, **f**) PD. The numbers of the cases in each group are the same as in Fig 1.

The sequential changes in perforin levels were not different between the PR, SD, and PD groups with nivolumab or pembrolizumab treatment (Fig 4). In the PD group, some factors showed significantly higher scores, which were associated with immune‐related adverse events, such as dermatitis or hepatitis.

**Figure 5:**
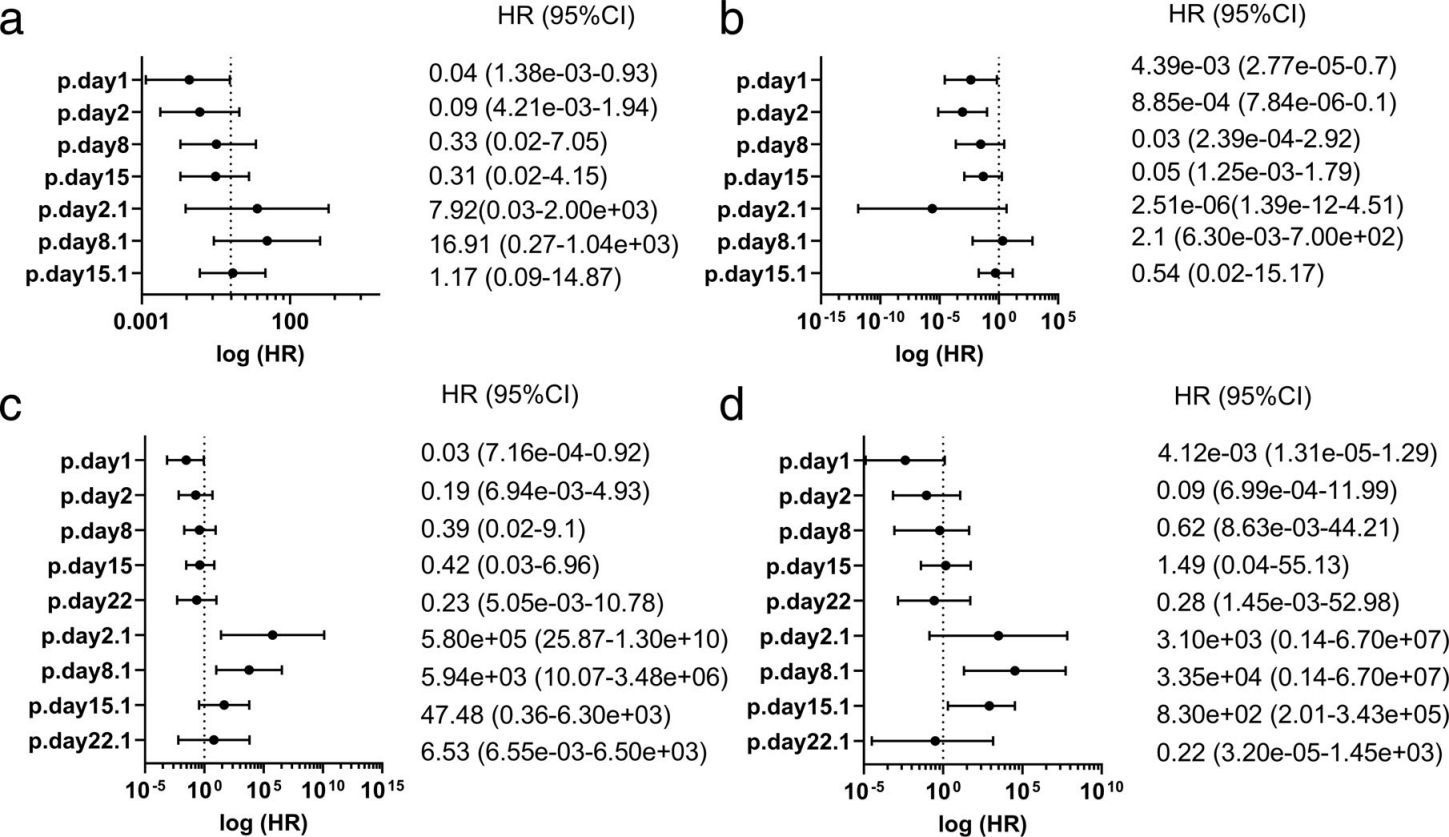
Analysis of risk factors for PFS and OS according to serum perforin concentration. The plot of the HR for PFS and OS, according to the serum perforin concentration at different time points or the ratio of the serum perforin concentration at different time points divided by the baseline serum perforin concentration. (**a**, **b**) Nivolumab and (**c**, **d**) pembrolizumab. (**a**, **c**) PFS, (**b**, **d**) OS. p., perforin; p.day2.1, p.day2/p.day1; p.day8.1, p.day8/p.day1; p.day15.1, p.day15/p.day1; p.day22.1, p.day22/p.day1.

The benefit of anti‐PD‐1 antibody therapies with respect to PFS or OS was evident in baseline perforin levels (p.day1; Fig 5). In sequential changes, the benefit of nivolumab with regard to prognosis was not clear (Fig 5a,b), but that of pembrolizumab was apparent on ratios of day 2/day 1 and day 8/day 1 (p.day2.1, p.day8.1; Fig 5c,d).

When cases were divided into the PR versus SD + PD groups, with nivolumab, the AUC at a ratio of day 15/day 1 was highest, but not sufficient, and considered to have low predictive power (Table 3A). With pembrolizumab, the AUC at a ratio of day 22/day 1 was higher than 0.7 but was not significant by log‐rank test or Cox regression analysis, which indicates that the value was appropriate to predict the best response but not the prognosis (Table 3B).

**Table 3:** The cutoff levels of perforin for PFS and OS by Cox proportional hazards model

**(a) Nivolumab, PR versus SD + PD**							
			**PFS**			**OS**		
	**Cutoff**	**AUC**	**Coefficients**	**HR (95% CI)**	***P***	**Coefficients**	**HR (95% CI)**	***P***
p.day2.1	0.867	0.504	−3.419	0.03 (4.52e‐03‐0.24)	0.001*	−3.147	0.04 (6.94e‐03‐0.27)	0.001*
p.day8.1	0.687	0.568	−2.89	0.06 (5.04e‐03‐0.61)	0.018*	−2.614	0.07 (7.52e‐03‐0.71)	0.024*
p.day15.1	0.454	0.622	−0.652	0.52 (0.12–2.26)	0.384	−0.807	0.45 (0.06–3.58)	0.448
**(b) Pembrolizumab, PR versus SD + PD**								
			**PFS**			**OS**		
	**Cutoff**	**AUC**	**Coefficients**	**HR (95% CI)**	***P***	**Coefficients**	**HR (95% CI)**	***P***
p.day2.1	0.743	0.521	0.335	1.4 (0.18–10.68)	0.747	18.191	7.95e+07(0.00e+00‐inf)	0.998
p.day8.1	0.903	0.588	0.889	2.43 (0.32–18.62)	0.392	0.267	1.31(0.16–10.49)	0.802
p.day15.1	1.204	0.513	0.758	2.13 (0.75–6.11)	0.158	1.398	4.05(0.96–17.1)	0.057
p.day22.1	1.185	0.714	0.172	1.19 (0.4–3.55)	0.758	−0.321	0.73(0.16–3.26)	0.675
**(c) Nivolumab, PR + SD versus PD**								
			**PFS**			**OS**		
	**Cutoff**	**AUC**	**Coefficients**	**HR (95% CI)**	***P***	**Coefficients**	**HR (95% CI)**	***P***
p.day2.1	0.872	0.548	3.014	0.05 (9.63e‐03‐0.25)	0*	3.626	0.03 (4.11e‐03‐0.17)	0*
p.day8.1	1.223	0.674	0.604	1.83 (0.67–4.99)	0.238	0.271	1.31 (0.28–6.12)	0.73
p.day15.1	1.416	0.6	0.394	1.48 (0.5–4.44)	0.481	−19.273	4.27e‐09 (0.00e+00‐inf)	0.999
**(d) Pembrolizumab, PR + SD versus PD**								
			**PFS**			**OS**		
	**Cutoff**	**AUC**	**Coefficients**	**HR (95% CI)**	***P***	**Coefficients**	**HR (95% CI)**	***P***
p.day2.1	1.086	0.647	0.864	2.37 (0.83–6.76)	0.106	0.76	2.14 (0.57–8.08)	0.262
p.day8.1	1.51	0.66	2.788	16.26 (2.23–1.18e+02)	0.006*	2.788	16.26 (2.23–1.18e+02)	0.006*
p.day15.1	0.558	0.46	−1.337	0.26 (0.03–2.19)	0.217	8.092	7.20e+07 (0.00e+00‐inf)	0.999
p.day22.1	0.972	0.611	−1.985	0.14 (0.03–0.57)	0.006*	−1.388	0.25 (0.06–1.12)	0.07

* Indicates *P* < 0.05.

AUC, area under the receiver operating characteristic curve; CI, confidence interval; HR; hazard ratio; OS, overall survivial; p. perforin; p.day2.1, p.day2/p.day1; p.day8.1, p.day8/p.day1; p.day15.1, p.day15/p.day1; p.day22.1, p.day22/p,day1; PD, progressive disease; PFS, progression‐free survival; PR, partial response; SD, stable disease.

When cases were divided into the PR + SD versus PD groups, for both nivolumab and pembrolizumab, all AUCs were approximately 0.6 and considered to have low predictive power (Table 3C,D).

## Discussion

In this study, we evaluated the changes in serum immune‐modulating factors as biomarkers of the response during the first cycle of anti‐PD‐1 antibody therapy in patients with advanced NSCLC. We demonstrated a statistically significant longer PFS and OS for patients with higher baseline concentrations of perforin than those with lower baseline concentrations.

Perforin is a pore‐forming protein in the cell membranes of target cells.^1^, ^16^, ^17^ Through the recognition of target cells via T cell receptor interactions, perforin is released from the secretory granules of cytotoxic lymphocytes together with proapoptotic serine protease granzymes. Following the exocytic release, monomeric perforin is oligomerized into membrane‐spanning pores at neutral pH and in the presence of Ca^2+^. After the pores are formed, granzyme B diffuses in the target cell cytosol. Once granzyme B is internalized into the target cells, granzyme B activates apoptosis in a caspase‐dependent manner.

Perforin and granzyme B exist mainly in cytotoxic lymphocytes, such as cytotoxic T cells or natural killer cells. Perforin and granzyme B also exist extracellularly in blood serum and plasma. There are only limited reports about the serum concentrations of perforin associated with pathophysiological status.^18^, ^19^, ^20^, ^21^, ^22^, ^23^, ^24^ In our study, perforin, but not granzyme B, was measured by ELISA. The serum concentration of perforin is not proportional to that of granzyme B.^18^ The perforin concentration may be much higher than the granzyme B concentration to be detected by ELISA, which might be explained by perforin being more stable than granzyme B in the extracellular space.

In healthy subjects, the perforin concentration is approximately 10 ng/mL.^23^ In the present study, the baseline concentration of perforin in lung cancer patients was approximately 5–6 ng/mL, which indicates that serum perforin concentration is low in lung cancer patients. These results are in agreement with those of a previous study that used flow cytometry and showed that the percentage of T cells, NK‐like T cells, and NK cells expressing intracellular perforin and granzyme B are lower in lung cancer tissue than in non‐lung‐cancer tissue.^25^ Although there are differences between intra‐ and extracellular perforin, its expression is low in lung cancer patients.

A complete deficiency of perforin results in an abnormal immune disorder known as familial hemophagocytic lymphohistiocytosis (HLH), whereas partial deficiency of perforin induces late‐onset HLH or hematological malignancies.^1^, ^26^, ^27^ Impaired perforin production results in extreme T cell activation, which leads to fatal inflammatory disorders such as HLH. A feedback loop to balance immune homeostasis is based on (i) T cells being activated by antigen presentation via antigen‐presenting dendritic cells (DCs); and (ii) activated CD8^+^ T cells selectively eradicating DCs that continue to present antigens. Perforin plays a role in eliminating selective antigen‐presenting DCs.^28^ In lung cancer, the cytotoxic activity produced by T cell activation is insufficient, and perforin levels may be kept low via this negative feedback loop.

Our study showed that there were differences between nivolumab and pembrolizumab with regard to (i) the optimal cutoff values for the baseline concentration; and (ii) the benefit on prognosis in sequential changes. As we have noted, there are limited studies about the serum perforin concentration associated with malignancies. The differences might be because of (i) the differences between nivolumab and pembrolizumab^29^; or (ii) patients with no previous treatment with chemotherapies were included in pembrolizumab group.

PD‐L1 expression in the tumor assessed by IHC is closely associated with prognosis.^9^, ^10^, ^11^ Patients whose tumors have high expression levels of PD‐L1 have improved clinical outcomes. We studied the correlation between the baseline perforin concentration and PD‐L1 protein expression in the tumor, and our scatterplot data showed that there was no considerable correlation. In contrast, perforin expression by quantitative reverse transcriptase‐polymerase chain reaction (RT‐PCR) in the tumor was significantly correlated with PD‐L1.^30^ These differences might be because we assessed different tumor types, different sites of perforin expression, or differences of perforin mRNA/protein expression.

Tumors with *EGFR* mutations and ALK rearrangements have low response rates to anti‐PD‐1/anti‐PD‐L1 antibodies.^12^, ^13^, ^14^, ^15^ The weak immunogenicity of the tumor microenvironment in these tumors might be due to a low tumor mutation burden or the low number of CD8^+^ tumor‐infiltrating lymphocytes. Our study showed that nivolumab treatment produced lower perforin levels in mutated EGFR cases; however, these levels were not significantly different between patients with or without *EGFR* mutations. This observation implies that factors other than *EGFR* mutations might be involved in regulating the CD8^+^ T cell activation loop associated with perforin. Moreover, we should note that in the present study, one patient who had low baseline perforin levels and *EGFR* mutations had a good prognosis. NSCLC with tyrosine kinase receptor mutations may have aberrant CD8^+^ T cell activation.

By PD‐1/PD‐L1 blockade, exhausted CD8^+^ T cells become reinvigorated and release immune modulators, which induce antitumor activity. CD8^+^ T cells in responsive patients might be more likely to release immune modulators than those in nonresponsive patients. In support of this hypothesis, we found that some sequential changes in perforin levels after nivolumab or pembrolizumab treatment contributed to a favorable prognosis. However, by calculating the optimal cutoff levels in efficacy with a Cox regression analysis, the values of these sequential changes were considered either to have low predictive power or no association with prognosis. Thus, the baseline perforin level is more appropriate than sequential perforin changes to predict the prognosis of NSCLC patients treated with anti‐PD‐1 antibody therapy.

There are certain limitations to this study. First, this was a small cohort, and we could not conclude the best cutoff levels of perforin to determine prognosis. Nevertheless, we were able to show that increased baseline perforin levels were strongly associated with prognosis. Second, we studied 11 immune modulators, which were highly selective and therefore had only limited results. More relevant candidate factors may exist to predict the clinical outcome of anti‐PD‐1 antibody therapy.

In summary, this study investigated serum immune modulators during the first cycle of anti‐PD‐1 antibody treatment in NSCLC. Measuring serum perforin levels before treatment may predict the clinical outcome of NSCLC patients treated with anti‐PD‐1 antibody therapy, which might be beneficial.

## Disclosures

Dr Nakahara reports grants and personal fees from Ono Pharmaceutical Co. Ltd., Bristol‐Myers Squibb K.K. and Eli Lilly Japan K.K., grants from Takeda Pharmaceutical Co., Ltd. and personal fees from AstraZenaka K.K., Chugai Pharmaceutical Co., Ltd., Taiho Pharmaceutical Co., Ltd. and Nippon Boehringer Ingelheim Co., Ltd. outside the submitted work. Dr Fukuoka reports personal fees from Eisai Co., Ltd. and Chugai Pharmaceutical Co., Ltd. outside the submitted work.

All other authors have nothing to declare.

## Supporting information


**Figure S1** Serum baseline CD137 levels and efficacy of nivolumab and pembrolizumab. (A, B) Baseline levels of CD137 are plotted by efficacy. All cases were measured by ELISA. The results are the medians, and the whiskers are the minimum to maximum. (A) Nivolumab and (B) pembrolizumab. (A) PR, *n* = 6; SD, *n* = 6, PD, *n* = 6. Data were analyzed by ANOVA. PR versus SD, *P* = 0.9983; PR *vs*. PD, *P* = 0.4388; SD versus PD, *P* = 0.4692. (B) PR, *n* = 4; PD, n = 3. Data were analyzed by Student's *t*‐test. PR versus, *P* = 0.1470. (C‐G) Sequential changes in CD137 levels with nivolumab or pembrolizumab treatment. The Y‐axis shows the ratio of the serum perforin concentration at each time point divided by the baseline serum perforin concentration. (C–E) Nivolumab and (F, G) pembrolizumab. (C, F) PR, (D) SD, (E, F) SD. (C) *n* = 7, (D) *n* = 7, (E) *n* = 7, (F) *n* = 6, (G) *n* = 5.
**Figure S2.** Receiver operating characteristic (ROC) curves of baseline concentration of perforin predicting the clinical outcome by anti‐PD‐1 antibody therapies. (A) Nivolumab and (B) pembrolizumab. MCT: misclassification‐cost term.
