# Supplementary material for: Serum immune modulators during the first cycle of anti‐PD‐1 antibody therapy in non‐small cell lung cancer: Perforin as a biomarker
Source: Thorac Cancer. 2020 Sep 11;11(11):3223–33. doi: 10.1111/1759-7714.13650 (PMC7606020; doi:10.1111/1759-7714.13650)
Supplement: Supplementary file 1 — Figure S1 Serum baseline CD137 levels and efficacy of nivolumab and pembrolizumab. (A, B) Baseline levels of CD137 are plotted by efficacy. All cases were measured by ELISA. The results are the medians, and the whiskers are the minimum to maximum. (A) Nivolumab and (B) pembrolizumab. (A) PR, n = 6; SD, n = 6, PD, n = 6. Data were analyzed by ANOVA. PR versus SD, P = 0.9983; PR vs. PD, P = 0.4388; SD versus PD, P = 0.4692. (B) PR, n = 4; PD, n = 3. Data were analyzed by Student's t‐test. PR versus, P = 0.1470. (C‐G) Sequential changes in CD137 levels with nivolumab or pembrolizumab treatment. The Y‐axis shows the ratio of the serum perforin concentration at each time point divided by the baseline serum perforin concentration. (C–E) Nivolumab and (F, G) pembrolizumab. (C, F) PR, (D) SD, (E, F) SD. (C) n = 7, (D) n = 7, (E) n = 7, (F) n = 6, (G) n = 5. Figure S2. Receiver operating characteristic (ROC) curves of baseline concentration of perforin predicting the clinical outcome by anti‐PD‐1 antibody therapies. (A) Nivolumab and (B) pembrolizumab. MCT: misclassification‐cost term. [file TCA-11-3223-s001.docx]

**

Supplementary Figure 1.** Serum baseline CD137 levels and efficacy of nivolumab and pembrolizumab. (A, B) Baseline levels of CD137 are plotted by efficacy. All cases were measured by ELISA. The results are the medians, and the whiskers are the minimum to maximum. (A) Nivolumab and (B) pembrolizumab. (A) PR, n=6; SD, n=6, PD, n=6. Data were analyzed by ANOVA. PR vs. SD, p=0.9983; PR vs. PD, p=0.4388; SD vs. PD, p=0.4692. (B) PR, n=4; PD, n=3. Data were analyzed by Student’s t-test. PR vs. PD, p=0.1470. (C-G) Sequential changes in CD137 levels with nivolumab or pembrolizumab treatment. The Y-axis shows the ratio of the serum perforin concentration at each time point divided by the baseline serum perforin concentration. (C-E) Nivolumab and (F, G) pembrolizumab. (C, F) PR, (D) SD, (E, F) SD. (C) n=7, (D) n=7, (E) n=7, (F) n=6, (G) n=5.





**Supplementary Figure 2.** Receiver operating characteristic (ROC) curves of baseline concentration of perforin predicting the clinical outcome by anti-PD-1 antibody therapies. (A) Nivolumab and (B) pembrolizumab. MCT: misclassification-cost term.
